# Comparison of the Effects of Cold-Water Immersion Applied Alone and Combined Therapy on the Recovery of Muscle Fatigue After Exercise: A Systematic Review and Meta-Analysis

**DOI:** 10.3390/life15081205

**Published:** 2025-07-28

**Authors:** Junjie Ma, Changfei Guo, Long Luo, Xiaoke Chen, Keying Zhang, Dongxue Liang, Dong Zhang

**Affiliations:** 1 Institute of Artificial Intelligence in Sports, Capital University of Physical Education and Sports, Beijing 100191, China; 18571611813@163.com (J.M.); gcf08182024@163.com (C.G.); 2 Department of Physical Education and Research, Central South University, Changsha 410083, China; 8104220108@csu.edu.cn; 3 Department of Physical Education, Tsinghua University, Beijing 100084, China; chenxk678@163.com; 4 Department of Physical Education, Southeast University, Nanjing 210096, China; bsuzky0812@163.com

**Keywords:** cold-water immersion, cold-water combination therapy, muscle fatigue, meta-analysis, delayed muscle soreness

## Abstract

Cold-water immersion (CWI), as a common recovery method, has been widely used in the field of post-exercise fatigue recovery. However, there is still a lack of comprehensive and systematic scientific evaluation of the combined effects of cold-water immersion combined with other therapies (CWI + Other). The aim of this study was to compare the effects of CWI and CWI + Other in post-exercise fatigue recovery and to explore the potential benefits of CWI + Other. We systematically searched PubMed, Embase, Web of Science, Cochrane Library and EBSCO databases to include 24 studies (475 subjects in total) and performed a meta-analysis using standardized mean difference (SMD) and 95% confidence intervals (CIs). The results showed that both CWI + Other (SMD = −0.68, 95% CI: −1.03 to −0.33) and CWI (SMD = −0.37, 95% CI: −0.65 to −0.10) were effective in reducing delayed-onset muscle soreness (DOMS). In subgroup analyses of athletes, both CWI + Other (SMD = −1.13, 95% CI: −1.76 to −0.49) and CWI (SMD = −0.47, 95% CI: −0.87 to −0.08) also demonstrated significant effects. In addition, CWI + Other significantly reduced post-exercise C-reactive protein (CRP) levels (SMD = −0.62, 95% CI: −1.12 to −0.13), and CWI with water temperatures higher than 10 °C also showed a CRP-lowering effect (MD = −0.18, 95% CI: −0.30 to −0.07), suggesting a potential benefit in anti-inflammation. There were no significant differences between the two interventions in the metrics of creatine kinase (CK; CWI: SMD = −0.01, 95% CI: −0.27 to 0.24; CWI + Other: SMD = 0.26, 95% CI: −0.51 to 1.03) or countermovement jump (CMJ; CWI: SMD = 0.22, 95% CI: −0.13 to 0.57; CWI + Other: SMD = 0.07, 95% CI: −0.70 to 0.85).

## 1. Introduction

Muscle fatigue (MF) is a transient decrease in the maximal voluntary-force-generating capacity of skeletal muscle induced by exercise. Its occurrence is closely related to insufficient neuromuscular drive, peripheral muscle metabolic disturbances or structural microdamage [1]. This reversible physiological process not only limits exercise performance but also increases the risk of exercise-related injuries by inducing tissue inflammation, delayed-onset muscle soreness (DOMS) and perceived fatigue [2]. The indicators of muscle fatigue after exercise are multidimensional, with physiological markers including creatine kinase (CK), C-reactive protein (CRP), lactate dehydrogenase (LDH) and interleukin-6 (IL-6), all of which serve as markers of injury and inflammation [3,4]. Changes in muscle contractile function are often assessed through kinetic tests, such as the countermovement jump (CMJ) [5,6]. These metrics are essential for evaluating both the extent of exercise-induced fatigue and the efficacy of recovery strategies.

Currently, extensive research has investigated diverse techniques for muscle fatigue recovery, including manual massage [7], vibration therapy [7,8,9], low-intensity restorative exercise [10], nutritional interventions [11] and cold therapy [12], all aimed at optimizing the recovery of muscle function. In recent years, cryostimulation has gained attention as a recovery technique to improve athletic performance and enhance post-exercise recovery [4]. Cryotherapy, which includes ice, cold water and cold air, is popular for its ability to remove body heat, lower core and tissue temperatures and alter blood flow [13]. Common cryotherapy methods include cold-water immersion (CWI), cold packs and ice massage [14]. These modalities can reduce inflammation and edema, improve blood circulation, provide analgesia and alleviate muscle spasms [15]. As a common form of cold therapy, CWI has garnered significant attention in competitive sports due to its ease of use and notable recovery benefits [16]. The available evidence suggests that CWI positively impacts muscle strength, soreness, serum CK levels and subjective recovery perception within the first 24 h after exercise [17,18]. However, there is significant heterogeneity in CWI implementation parameters and the populations in which it is applied, and its effects on muscle damage markers, relief of soreness and the rate of strength recovery remain inconclusive.

Although meta-analyses have been conducted to investigate the effects of CWI on post-exercise fatigue recovery [16], there is a lack of systematic quantitative analysis comparing the effects of CWI vs. cold-water immersion combined with other therapies (CWI + Other) (e.g., compression therapy, nutritional supplementation and massage) in different sport populations. In view of the paucity of original studies allowing for direct comparisons, indirect comparisons were made by means of blank controls of similar conditions. The aim was to fill this gap by integrating existing evidence through meta-analysis. Specifically, passive recovery (e.g., sedentary rest or placebo) was used as the benchmark comparator, representing an unintervened, natural recovery process. Utilizing this no-intervention state as a baseline allowed for the isolation and measurement of the net therapeutic effect of CWI and CWI + Other, thereby quantifying the true efficacy of the treatments against natural recovery. The study sought to clarify (1) whether CWI + Other is more effective than CWI for muscle fatigue recovery; (2) the differences in the effects of these two interventions on muscle fatigue recovery in athletes vs. non-athletes to determine which intervention is superior; and (3) the quantitative and qualitative effects on muscle damage markers (CK, CRP), soreness (DOMS) and recovery of countermovement jump (CMJ). The results of this study will provide an evidence-based foundation for precise skeletal muscle fatigue recovery after exercise and offer scientific guidance for athletes in selecting the appropriate CWI intervention and core parameters in practice.

## 2. Materials and Methods

The protocol for this systematic review was registered with the International Prospective Register of Systematic Reviews (PROSPERO), registration number CRD420251007507.

### 2.1. Search Strategy

The systematic review followed the four stages outlined in the PRISMA guidelines: identification, screening, eligibility assessment and inclusion. A comprehensive search of PubMed, Embase, Web of Science, EBSCO and Cochrane databases was conducted to evaluate the effects of CWI and CWI + Other on the recovery of muscle fatigue, up to 9 March 2025. Only English-language literature was included. The search strategy was adapted for the specific requirements of each database. The complete search strategy for each database is provided in Supplemental File S1.

### 2.2. Study Selection

Two reviewers (MJJ and GCF) first eliminated duplicates by leveraging EndNote X9 software. Subsequently, they independently scanned the titles and abstracts to identify potentially relevant studies. Any discrepancies that arose during this process were addressed through discussion with a third reviewer (ZD). For studies that met the inclusion criteria, two reviewers independently conducted assessments. The inclusion criteria for studies were as follows: (1) participants had to be healthy adults, without serious illness or disability, and aged between 13 and 50 years; (2) the interventions included CWI or CWI + Other, where CWI + Other specifically included CWI combined with compression, taking vitamins, massage and active exercise. The modality, duration and temperature of the interventions were recorded in detail; (3) the control groups consisted of passive recovery, sedentary rest or placebo; (4) the outcome metrics included at least one of the following: validated tests and measurements of CK, DOMS, CRP and CMJ; (5) the study design had to include a randomized controlled trial, a randomized crossover trial or a quasi-randomized controlled trial or any intervention-based trials. The reasons for study exclusion included (1) duplicated or missing data; (2) the type of intervention did not include CWI; (3) the outcome measures did not include any of the listed variables; (4) authorship was unknown, or the article title did not match; (5) the article was not written in English.

### 2.3. Data Extraction

Two reviewers (MJJ and GCF) independently extracted data from the included studies, and any inconsistencies were resolved through discussion with a third reviewer (ZD). The data extracted included the year of publication, type of study, fatigue induction protocol, outcome indicators (CK, DOMS, CRP and CMJ), form of intervention (CWI, CWI + Other) and details regarding the experimental and control groups (number of participants, occupation and age).

### 2.4. Quality Assessment

The risk of bias for each included study was assessed by two pairs of authors (MJJ and GCF; MJJ and ZD) using the first version of the Cochrane risk-of-bias tool for randomized controlled trials (RoB 1) [19]. This tool is detailed in Chapter 8 of the *Cochrane Handbook for Systematic Reviews of Interventions* [19] and was selected for its comprehensive domain-based evaluation. Due to the nature of exercise interventions, it was deemed impractical to blind participants; therefore, only six types of bias were assessed, including random sequence generation, allocation concealment, blinding of outcome assessors, incomplete outcome data, selective outcome reporting and other potential risks of bias.

### 2.5. Sensitivity Analysis

To ensure the robustness of the findings, sensitivity analyses were employed. This study initially included 25 studies for qualitative synthesis. However, one study, which only provided data for the CK outcome, was excluded from all quantitative analyses prior to pooling due to significant methodological heterogeneity. Before exclusion, the effect size for CK was SMD = −0.10 (95% CI: −0.33 to 0.14, I^2^ = 69%), and after exclusion, the heterogeneity was substantially reduced (I^2^ = 11%). It was hypothesized that the high heterogeneity could be attributed to the experimental group in this study performing CWI for 9 consecutive days, whereas most other studies involved single or short-term interventions. This resulted in a final set of 24 studies for the meta-analysis.

Furthermore, for specific outcomes within this set of 24 studies, additional sensitivity analyses were conducted where high heterogeneity was present. Notably, for the DOMS outcome, one study (Sánchez-Ureña, 2017) [20] was identified as an outlier and was excluded from the DOMS-specific analysis to reduce heterogeneity, although its data for other outcomes (e.g., CMJ) were retained. This process is detailed in the corresponding Section 3.

### 2.6. Statistical Analysis

Data extraction involved calculating the change in mean and standard deviation (SD) before and after the intervention. Statistical analyses were conducted using Review Manager (RevMan) [Computer program], Version 5.3, The Cochrane Collaboration, 2014. After determining the overall effect size, subgroup analyses were conducted based on subject occupation (athletes vs. non-athletes) and water temperature (below or above 10 °C). One study further subdivided athletes into three subcategories: (1) elite athletes exercising ≥10 h per week and competing at the highest level; (2) competitive athletes exercising ≥6 h per week, focusing on performance improvement and formal competition; and (3) recreational athletes exercising ≥4 h per week, primarily for fitness or informal competition. Non-athletes were defined as individuals performing ≥2.5 h of physical activity per week, mainly to maintain health and fitness [21]. In this study, athletes and non-athletes were classified based on the above criteria. The significance level was set at 0.05. Effect sizes were calculated as mean differences (MDs) with 95% confidence intervals (CIs) when outcome measures were reported using the same units (e.g., CRP). When different units were used for the same outcome (e.g., DOMS), standardized mean differences (SMDs) were calculated instead to allow for pooling. Statistical heterogeneity among studies was quantified using the I^2^ statistic, with values of 25%, 50% and 75% representing low, moderate and high heterogeneity, respectively. A fixed-effects model was initially chosen for data pooling; however, a random-effects model was employed in cases of moderate-to-high heterogeneity I^2^ > 50% to account for between-study variance. To explore potential sources of heterogeneity, sensitivity analyses were conducted by systematically removing one study at a time.

To further investigate potential sources of heterogeneity, meta-regression analyses were conducted to test the influence of study-level covariates, including total sample size and mean participant age. These analyses were restricted to the CWI-only subgroup for outcomes with moderate-to-high heterogeneity, as the CWI + Other group had an insufficient number of studies for a reliable regression.

## 3. Results

### 3.1. Included Studies

**Figure 1:**
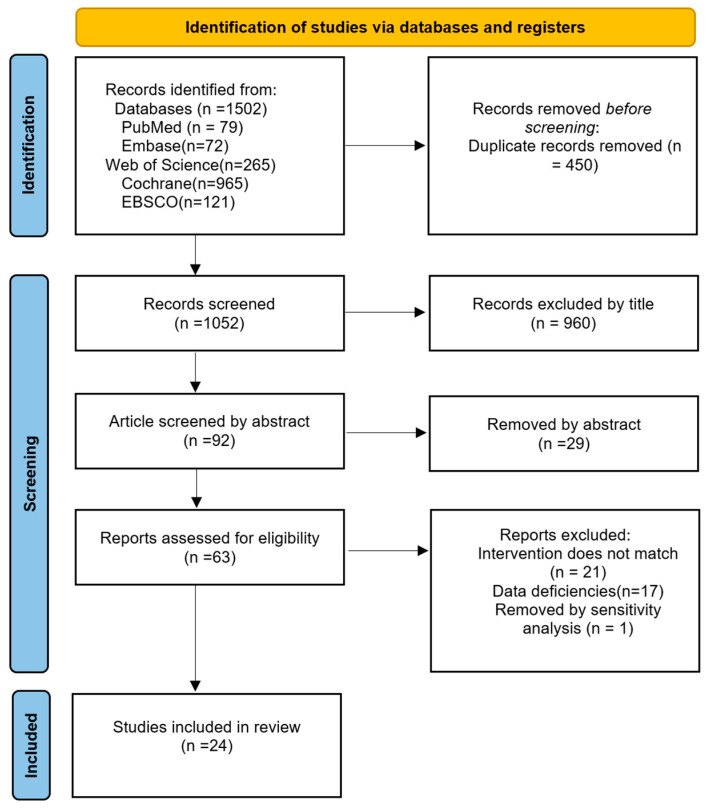
The flowchart illustrating the process of literature search and screening.

After conducting a comprehensive search across several databases, a total of 1502 articles were identified (PubMed: 79; Embase: 72; Web of Science: 265; Cochrane: 965; EBSCO: 121). Of these, 450 articles were excluded due to duplication or unknown authors. After reviewing the titles and abstracts, 63 articles remained. Upon reading the full texts, 38 articles were excluded, primarily because the intervention did not match the criteria (*n* = 21) or due to data deficiencies (*n* = 17). This resulted in 25 studies being included in the qualitative synthesis. As detailed in the Section 2.5, one of these studies was subsequently excluded from the quantitative synthesis due to high heterogeneity, leaving a final set of 24 studies included in the meta-analysis, as shown in Figure 1.

### 3.2. Description of the Included Trials

Table 1 summarizes the key characteristics of the included studies, including participant demographics, study design, fatigue induction modality, intervention modality and outcome indicators. A total of 475 participants were enrolled, with 18 studies focusing on CWI and 6 studies involving cold-water combination therapy. The gender distribution was uneven, with some studies not reporting the number of male and female participants in each group.

### 3.3. Risk of Bias Assessment

**Figure 2:**
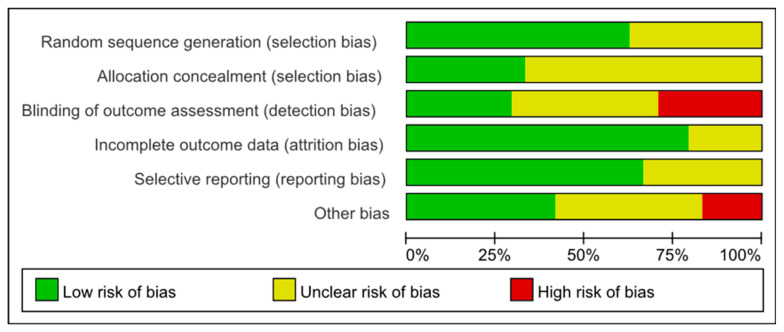
Overall quality assessment diagram of studies included in the research.

Figure 2 presents the risk-of-bias assessment for all the included articles using Cochrane’s risk-of-bias tool. It provides a summary of the quality assessment for each study, highlighting specific risks. Each article was evaluated on the six aspects: random sequence generation, allocation concealment, blinding of outcome assessment, incomplete outcome data, selective reporting and other sources of bias. Notably, seven articles had a sample size of fewer than 10 subjects, which may affect the reliability of the results due to the small sample size. Additionally, the lack of direct comparisons between cold-water immersion and cold-water immersion combination therapies means that the presented results were based on indirect comparisons between the experimental and control groups.

### 3.4. Synthesis of the Results

#### 3.4.1. CK

**Figure 3:**
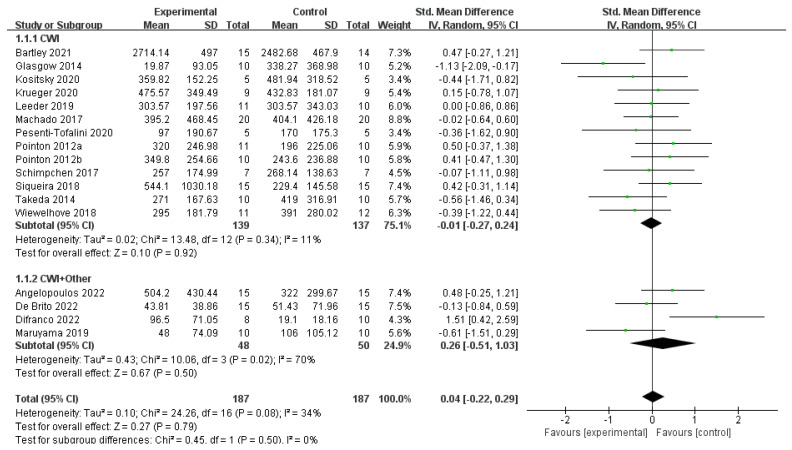
Forest plot of the effect of the meta-analysis of creatine kinase (CK) levels for CWI and CWI + Other interventions compared to a control group [22,25,26,29,30,32,33,34,35,36,38,39,40,41,42,43,44]. Each point represents the standardized mean difference (SMD) and its 95% confidence interval (CI) for an individual study. CWI = cold-water immersion, CWI + Other = cold-water immersion combined with other therapies. The “a” and “b” in Pointon 2012 are used to distinguish between two different articles published by the same author in the same year.

**Figure 4:**
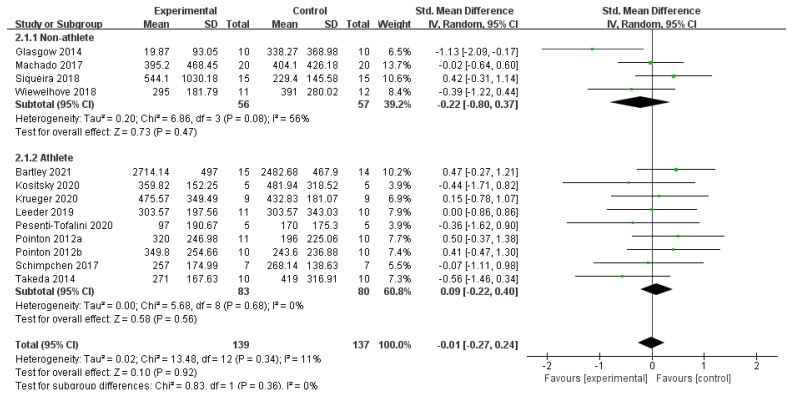
Forest plot of the subgroup analysis of creatine kinase (CK) levels for the CWI intervention, stratified by occupation (athlete vs. non-athlete) [22,26,29,32,33,34,35,36,39,40,41,42,44]. Each point represents the standardized mean difference (SMD) and its 95% confidence interval (CI) for an individual study. CWI = cold-water immersion. The “a” and “b” in Pointon 2012 are used to distinguish between two different articles published by the same author in the same year.

**Figure 5:**
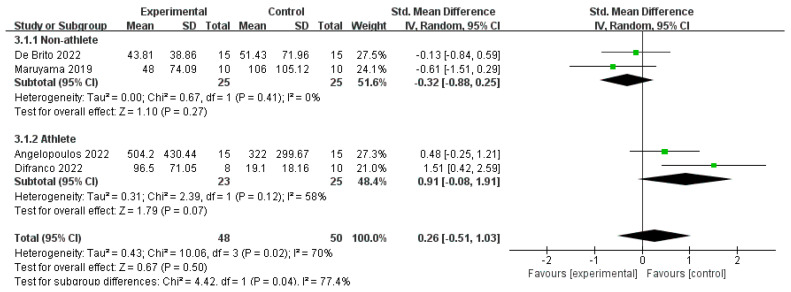
Forest plot of the subgroup analysis of creatine kinase (CK) levels for the CWI + Other intervention, stratified by occupation (athlete vs. non-athlete) [25,30,38,43]. Each point represents the standardized mean difference (SMD) and its 95% confidence interval (CI) for an individual study. CWI = cold-water immersion; CWI + Other = cold-water immersion combined with other therapies.

Based on 17 publications to determine whether post-exercise CWI and CWI + Other affected CK, the results showed no statistically significant difference between the CWI and CON groups (SMD = −0.01, 95% CI: −0.27 to 0.24, *p* = 0.92) and low between-study heterogeneity (I^2^ = 11%) (Figure 3). Furthermore, a subgroup analysis of the CWI group based on occupation showed no significant effects in either the non-athlete (SMD = −0.22, *p* = 0.47) or athlete subgroups (SMD = 0.09, *p* = 0.56), and no significant difference was found between these subgroups (*p* = 0.36) (Figure 4). For the CWI + Other intervention, no difference was statistically significant compared to the CON group (SMD = 0.26, 95% CI: −0.51 to 1.03, *p* = 0.50), with high inter-study heterogeneity (I^2^ = 70%). A further subgroup analysis was performed on this group, dividing it into athletes and non-athletes (Figure 5). The non-athlete subgroup showed non-significant and non-heterogeneous results (SMD = −0.32, 95% CI: −0.88 to 0.25, *p* = 0.27, I^2^ = 0%). In the athlete group (SMD = 0.91, 95% CI: −0.08 to 1.91, *p* = 0.07, I^2^ = 58%), a trend favoring the CON group was observed, but this did not reach statistical significance. Notably, the test for subgroup differences indicated a significant difference between the responses of athletes and non-athletes to the CWI + Other intervention (*p* = 0.04). This may be attributed to the fact that low temperatures can inhibit local blood flow, thereby causing a delay in the clearance of CK into the bloodstream and leading to a lag in the detection value.

#### 3.4.2. CMJ

**Figure 6:**
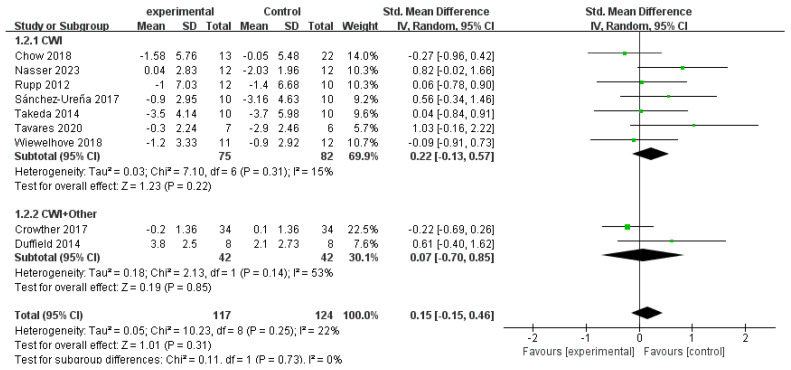
Forest plot of the meta-analysis of countermovement jump (CMJ) performance for CWI and CWI + Other interventions compared to a control group [20,23,24,27,28,31,36,37,44]. Each point represents the standardized mean difference (SMD) and its 95% confidence interval (CI) for an individual study. CWI = cold-water immersion; CWI + Other = cold-water immersion combined with other therapies.

The meta-analysis of CMJ performance revealed no statistically significant difference between the CWI group (seven studies) and the CON group (SMD = 0.22, 95% CI: −0.13 to 0.57, *p* = 0.22), with low heterogeneity observed (I^2^ = 15%) (Figure 6). Similarly, the CWI + Other group (two studies) showed no significant effect compared to the CON group (SMD = 0.07, 95% CI: −0.70 to 0.85, *p* = 0.85), although moderate heterogeneity was noted (I^2^ = 53%). The test for subgroup differences indicated no significant difference between the effects of the two intervention types (*p* = 0.73). The overall lack of a significant effect for either intervention may be attributed to the potential inhibitory effect of cold therapy on α-motor neuron excitability, which could negatively impact neural activation. Due to the limited number of included studies, particularly for the CWI + Other intervention, further subgroup analysis was not performed.

#### 3.4.3. DOMS

**Figure 7:**
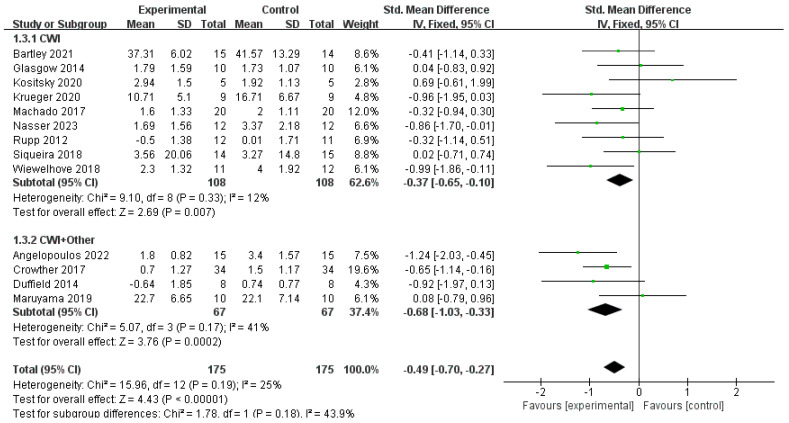
Forest plot of the meta-analysis of delayed-onset muscle soreness (DOMS) for CWI and CWI + Other interventions compared to a control group [20,22,24,27,29,31,32,33,35,37,38,42,43,44]. Each point represents the standardized mean difference (SMD) and its 95% confidence interval (CI) for an individual study. CWI = cold-water immersion; CWI + Other = cold-water immersion combined with other therapies.

Muscle soreness was assessed using different unit-based scales across 14 included studies; therefore, the standardized mean difference (SMD) was used for this analysis. For the CWI intervention, a sensitivity analysis was conducted by excluding one study (Sánchez-Ureña, 2017) [20], which was a source of high initial heterogeneity (I^2^ = 57%). The final analysis, based on the remaining studies, showed that CWI significantly reduced DOMS compared to the CON group (SMD = −0.37, 95% CI: −0.65 to −0.10, *p* = 0.007), with low final heterogeneity (I^2^ = 12%). In the CWI + Other group, the DOMS levels were also significantly lower compared to the CON group (SMD = −0.68, 95% CI: −1.03 to −0.33, *p* = 0.0002) (Figure 7). Although the SMD for CWI + Other (−0.68) was lower than for CWI (−0.37), the test for subgroup differences indicated no statistically significant difference between the effects of the two interventions (*p* = 0.18).

**Figure 8:**
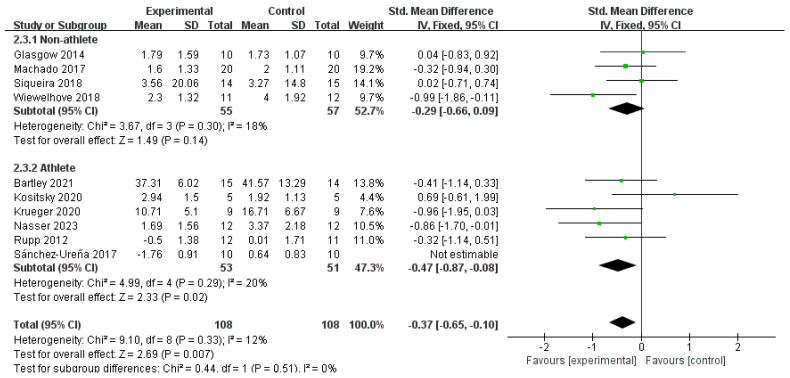
Forest plot of the subgroup analysis for the effect of the CWI intervention on delayed-onset muscle soreness (DOMS), stratified by occupation (athlete vs. non-athlete) [20,22,29,31,32,33,35,37,42,44]. Each point represents the standardized mean difference (SMD) and its 95% confidence interval (CI) for an individual study. CWI = cold-water immersion.

**Figure 9:**
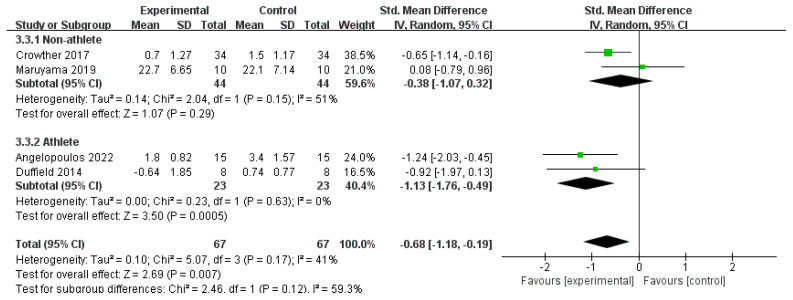
Forest plot of the subgroup analysis for the effect of the CWI + Other intervention on delayed-onset muscle soreness (DOMS), stratified by occupation (athlete vs. non-athlete) [24,27,38,43]. Each point represents the standardized mean difference (SMD) and its 95% confidence interval (CI) for an individual study. CWI = cold-water immersion; CWI + Other = cold-water immersion combined with other therapies.

Subgroup analyses were conducted based on athletes and non-athletes for both the CWI and CWI + Other groups (Figure 8 and Figure 9). For the CWI group, a significant effect on DOMS reduction was found in the athlete subgroup (SMD = −0.47, 95% CI: −0.87 to −0.08, *p* = 0.02) but not in the non-athlete subgroup (SMD = −0.29, 95% CI: −0.66 to 0.09, *p* = 0.14). The test for subgroup differences showed no significant difference between these two groups (*p* = 0.51). For the CWI + Other group, a random-effects model was used for the subgroup analysis due to moderate heterogeneity (I^2^ = 51%). The results indicated a significant effect in the athlete subgroup (SMD = −1.13, 95% CI: −1.76 to −0.49, *p* = 0.0005), while the effect in non-athletes was not statistically significant (SMD = −0.38, 95% CI: −1.07 to 0.32, *p* = 0.29). Similarly, there was no significant difference between the athlete and non-athlete subgroups for this intervention (*p* = 0.12).

To explore the sources of initial high heterogeneity within the CWI subgroup for the DOMS outcome, meta-regression analyses were conducted. The analyses revealed that neither total sample size (*p* = 0.887) nor mean participant age (*p* = 0.526) were significant moderators of the treatment effect. The bubble plots visualizing these analyses are available in the Supplemental File (see Supplemental Figures S1 and S2).

#### 3.4.4. CRP

**Figure 10:**
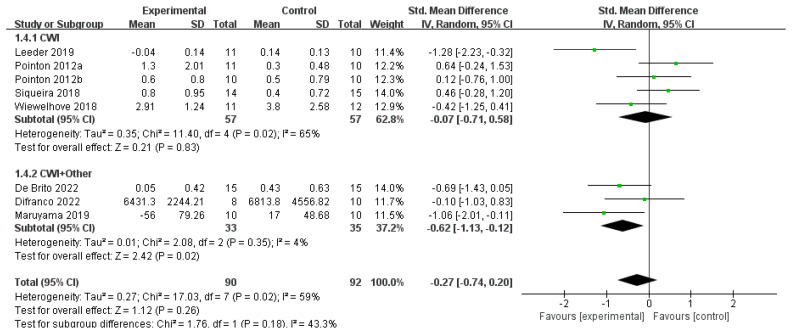
Forest plot of the meta-analysis of C-reactive protein (CRP) levels for CWI and CWI + Other interventions compared to a control group [25,30,34,39,40,42,43,44]. Each point represents the standardized mean difference (SMD) and its 95% confidence interval (CI) for an individual study. CWI = cold-water immersion; CWI + Other = cold-water immersion combined with other therapies. The “a” and “b” in Pointon 2012 are used to distinguish between two different articles published by the same author in the same year.

**Figure 11:**
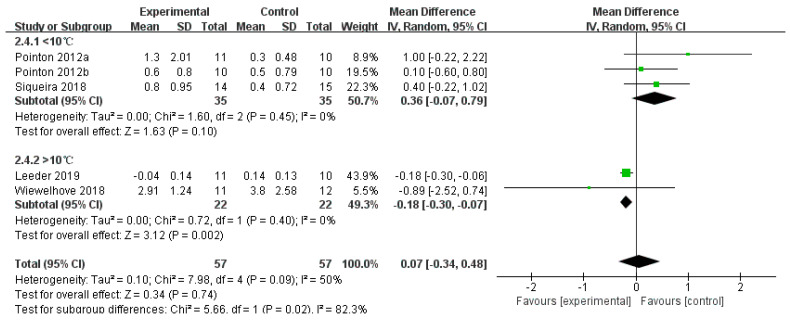
Forest plot of the subgroup analysis of the effect of the CWI intervention on C-reactive protein (CRP) levels, stratified by water temperature (<10 °C and >10 °C) [34,39,40,42,44]. Each point represents the standardized mean difference (SMD) and its 95% confidence interval (CI) for an individual study. CWI = cold-water immersion. The “a” and “b” in Pointon 2012 are used to distinguish between two different articles published by the same author in the same year.

A total of eight studies reported CRP levels. The main analysis, using a random-effects model, showed that for the CWI group, there was no statistically significant difference in CRP levels compared to the control group (SMD = −0.07, 95% CI: −0.71 to 0.58, *p* = 0.83). In contrast, the CWI + Other group showed a significant reduction in post-exercise CRP levels (SMD = −0.62, 95% CI: −1.13 to −0.12, *p* = 0.02), with low heterogeneity (I^2^ = 4%). However, the test for subgroup differences indicated no statistically significant difference between the effects of the two interventions (*p* = 0.18) (Figure 10). When CWI was divided based on temperature (below 10 °C and above 10 °C), the subgroup analysis revealed that temperatures below 10 °C did not have a significant effect on CRP (MD = 0.36, 95% CI: −0.07 to 0.79, *p* = 0.10) (Figure 11). In contrast, CWI with water temperatures above 10 °C resulted in a significant reduction in CRP levels (MD = −0.18, 95% CI: −0.30 to −0.07, *p* = 0.002). The test for subgroup differences confirmed that the effect was significantly different between the two temperature ranges (*p* = 0.02).

To statistically test the potential sources of the high heterogeneity found in the CWI group for the CRP outcome, a meta-regression analysis was performed for the influence of total sample size. As shown in the Supplemental File (see Supplemental Figure S3), the analysis revealed that total sample size was not a significant moderator of the treatment effect (*p* = 0.645). A similar analysis for mean participant age was not conducted, as an insufficient number of studies within the CWI subgroup reported these data (three out of five studies), precluding a reliable regression analysis.

## 4. Discussion

### 4.1. Summary of Evidence

The main findings of this study indicate that both CWI and CWI + Other are effective in reducing post-exercise delayed-onset muscle soreness (DOMS). Although the SMD for CWI + Other (−0.68) was lower than for CWI (−0.37), no significant difference was found between the two interventions (*p* = 0.18). Additionally, CWI + Other demonstrated more pronounced improvements in DOMS for athletes. Regarding inflammatory markers, CWI + Other significantly reduced CRP levels, whereas CWI only showed significant effects when the water temperature was above 10 °C, with the temperature difference being a significant factor. Furthermore, as shown in Figure 3 and Figure 6, the study found no significant differences between the two interventions in terms of recovery effects on CK and CMJ.

### 4.2. Comparison with Previous Studies

Compared to previous studies, the literature shows that CWI can effectively alleviate DOMS, which is consistent with the findings of Machado et al. [45]. Building on this, the present study also found that combining CWI with other recovery methods (such as compression garments, massage, active exercise and supplementation with vitamin C and cherry juice) resulted in a numerically larger effect size for reducing DOMS, suggesting a potential synergistic effect, although this difference did not reach statistical significance (*p* = 0.18). Compression garments, massage, active exercise and the intake of vitamin C/E and cherry juice have all been shown to reduce DOMS levels [25,30,38,43].

Regarding CRP, there is some discrepancy with previous research. Some studies have found no significant effect of CWI on CRP levels [16], while others have reported that CWI can reduce CRP levels [46]. In this study, it was found that CWI with water temperatures above 10 °C, as well as CWI + Other, can effectively reduce CRP levels.

### 4.3. Mechanism Discussion

#### 4.3.1. Core Mechanisms of CWI

The results of this study demonstrated that both CWI and CWI + Other significantly reduced delayed-onset muscle soreness (DOMS), with the CWI group showing an SMD of −0.37 (*p* < 0.05). The underlying mechanism is presumed to be closely associated with cold-induced neurovascular regulation. Studies have shown that cold environments activate TRPM8 cold receptors, which inhibit the firing frequency of cold-sensitive nerve endings and slow the transmission of nociceptive signals [47], which is consistent with the subjective reduction in DOMS observed after CWI.

Additionally, low temperatures suppress the Rho kinase and MEK signaling pathways, thereby reducing vascular smooth muscle contractility and decreasing peripheral vascular resistance [48], resulting in diminished local blood flow. This reduction in perfusion may inhibit the release of inflammatory mediators such as CRP, thereby alleviating tissue hematoma and pain signal transmission. Subgroup analysis revealed that CWI had a more pronounced effect on reducing DOMS in athletes (SMD = −0.47, *p* = 0.02), which may be attributed to the more intense inflammatory response and greater tissue microtrauma experienced after exercise in this population. Therefore, cold intervention may exert a more sensitive analgesic and anti-inflammatory effect following high-intensity physical activity.

#### 4.3.2. Synergistic Mechanisms of CWI + Other

Although the effect size of CWI + Other was larger for both DOMS and CRP, this difference was not statistically significant compared to CWI alone. The effect on CRP was significant, with an SMD of −0.62 (*p* = 0.02). The underlying mechanisms are presumed to involve synergistic effects from multidimensional interventions:

(1) Pressure-Assisted Effects of Compression Garments: Wearing compression garments following exercise can modulate osmotic pressure through external pressure gradients, thereby reducing swelling and hematoma formation [49]. When combined with the vasoconstrictive effect of CWI, this may further suppress inflammatory exudation. In this study, CWI + Other in the athlete subgroup achieved an SMD of −1.13 (*p* = 0.0005), notably larger than that of CWI (SMD = −0.47), suggesting that compression intervention may exert an additive inhibitory effect on acute inflammatory responses following high-intensity exercise.
(2) Antioxidant Synergy of Vitamins C and E: Vitamin C, a water-soluble antioxidant, scavenges cytoplasmic free radicals, while vitamin E, a lipid-soluble antioxidant, protects cell membranes from lipid peroxidation damage [25,50]. A combined supplementation can enhance antioxidant capacity through a regeneration cycle, thereby inhibiting NF-κB-mediated inflammatory responses [51]. In this study, the CRP-lowering effect of CWI + Other (SMD = −0.62) was consistent with that of CWI at water temperatures >10 °C (MD = −0.18), indicating that antioxidant supplementation may compensate for the incomplete suppression of inflammatory pathways by cold exposure alone, particularly in regulating biochemical markers less sensitive to temperature.
(3) Massage-Induced Local Blood Flow Enhancement: Massage promotes local circulation through mechanical pressure, alleviating vasoconstriction-induced ischemia caused by CWI, enhancing the delivery of oxygen and nutrients and simultaneously relaxing tight muscle fibers and fascia, thereby improving muscle extensibility [38]. This explains the larger observed effect of the combined intervention in relieving DOMS by achieving more effective pain relief and functional restoration through a multi-pathway synergy of “cold-induced analgesia, compression-mediated anti-edema and massage-facilitated circulation recovery”.

#### 4.3.3. Temperature-Dependent Mechanism of Inflammation Regulation

Our analysis confirmed a temperature-dependent mechanism, as CWI at water temperatures >10 °C significantly reduced CRP levels (MD = −0.18, *p* = 0.002), whereas temperatures <10 °C showed no effect. Importantly, this difference between the temperature subgroups was statistically significant (*p* = 0.02), strongly suggesting a threshold effect. This may be because temperatures above 10 °C are sufficient to activate an anti-inflammatory response without causing the excessive vasoconstriction that can occur at lower temperatures. When the water temperature exceeds 10 °C, the cold stimulus may be sufficient to activate TRPM8 receptor-mediated analgesic effects while avoiding excessive vasoconstriction that could lead to ischemia-induced inflammatory rebound. This helps maintain a balance between anti-inflammatory mediators (e.g., IL-10) and pro-inflammatory markers (e.g., CRP). In the CWI + Other group, CRP was reduced regardless of water temperature, indicating that adjunct interventions, such as antioxidant supplementation and massage, may overcome the limitations of temperature-dependent inflammatory regulation. These effects are likely mediated through temperature-independent pathways—such as direct free radical scavenging and inhibition of the NF-κB signaling cascade [51]—and are directly related to the oxidative-stress-reducing mechanisms of vitamin C and E supplementation [25].

#### 4.3.4. Potential Reasons for Non-Significant Effects on Certain Indicators

This study demonstrated that both CWI and CWI + Other had no statistically significant effects on CK levels or CMJ performance. One possible explanation is that CK is a delayed biomarker of muscle damage, and its levels are more susceptible to variability due to exercise type and the timing of post-exercise measurement. Specifically, CK levels typically peak between 24 and 72 h after exercise, while CRP, a marker of systemic inflammation, generally reaches its peak at approximately 24–48 h. The variability in measurement time points across the included studies means that some assessments may have captured these biomarkers before their peak, whereas others may have occurred during their decline. This potential timing bias could mask the true magnitude of muscle damage and inflammation, leading to an underestimation of the intervention’s efficacy and contributing to the non-significant findings observed in this meta-analysis. In contrast, CMJ reflects neuromuscular function, and the transient suppression of central drive caused by cold exposure may counteract any peripheral recovery benefits [1]. Future research should consider the cumulative effects of long-term interventions on CK as well as the potential of combined therapies to enhance neuromuscular coordination over extended recovery periods.

In conclusion, CWI alleviates DOMS primarily through neuroanalgesic and vasoconstrictive mechanisms, while combined interventions exert synergistic effects through pressure modulation, antioxidant action and mechanical stimulation. These mechanisms are particularly effective in athletic populations, resulting in enhanced anti-inflammatory and analgesic outcomes. Furthermore, the finding that CWI at water temperatures > 10 °C significantly reduces inflammatory markers provides mechanistic evidence to guide the optimized application of cold therapy in clinical and sports recovery settings.

### 4.4. Sources of Heterogeneity

This study identified substantial heterogeneity in the outcomes of DOMS and CRP. Upon reviewing the original literature, the heterogeneity was found to potentially stem from three main areas:

(1) Sample Size Variability: The studies exhibited a wide range of sample sizes, with experimental group sizes varying from 5 to 34 participants. To formally test whether this variability was a source of heterogeneity, we conducted meta-regression analyses. These analyses showed no significant association between sample size and the treatment effect for either the DOMS outcome (*p* = 0.887) or the CRP outcome (*p* = 0.645).
(2) Diverse Population Characteristics: The included studies comprised diverse populations, including both athletes and non-athletes, across all articles, with a broad age range (14–46 years old). To test the influence of age, a meta-regression was performed for the DOMS outcome, which found no significant association with the treatment effect (*p* = 0.526). A similar analysis for the CRP outcome was not feasible due to an insufficient number of studies reporting these data, preventing a conclusive determination of its influence on CRP heterogeneity.
(3) Exercise Protocols: The studies encompassed a variety of exercise types, including triathlon, basketball, high-intensity interval training (HIIT), tennis and soccer. These exercises differed in intensity and duration, leading to varying levels of exercise-induced fatigue. Such differences in exercise protocols can significantly impact the efficacy of CWI and CWI + Other, thereby contributing to the observed heterogeneity in recovery effects.

### 4.5. Limitations of the Study

Despite the valuable findings of this study, several limitations should be considered. Firstly, the search strategy for this review was focused on five major electronic databases and did not include a comprehensive search for gray literature, such as dissertations or conference proceedings. While this approach prioritizes peer-reviewed evidence, it is a limitation, as relevant studies not indexed in these databases may have been omitted. This could potentially impact the overall comprehensiveness of our evidence base. Secondly, the observed heterogeneity in some results, particularly for outcomes such as DOMS and CRP, suggests variability in study designs, populations and intervention protocols. This heterogeneity may be partly attributed to the omission of key subject information like age and gender in some studies, which can influence the generalizability of the conclusions drawn. Thirdly, the majority of included studies utilized different cold-water immersion temperatures and intervention durations, making it challenging to determine the optimal cold therapy parameters. Furthermore, the lack of long-term follow-up in most studies prevents the evaluation of sustained effects or potential adverse outcomes of CWI and combined interventions.

Additionally, this meta-analysis primarily relied on self-reported measures for DOMS, which could introduce bias and limit the objectivity of the findings. Furthermore, given the limited research in specific subgroups (e.g., non-athletic populations or individuals with different baseline fitness levels), further investigation into these subgroups is warranted to fully understand the broader applicability of CWI and CWI + Other interventions. Moreover, owing to the scarcity of studies providing direct comparisons between the two interventions (CWI and CWI + Other), we resorted to indirect comparisons by utilizing analogous control groups. This methodological choice may have influenced the reliability of the results.

Lastly, a formal assessment of publication bias using methods such as funnel plots or Egger’s test was not performed. This decision was based on recommendations from the *Cochrane Handbook* [19], which advises against such tests when fewer than 10 studies are included in a meta-analysis due to low statistical power and the risk of misleading results. As several of our key analyses, particularly those for the CMJ and CRP outcomes and for the CWI + Other subgroups, were based on a small number of studies, we determined that a formal publication bias analysis would be inappropriate and unreliable.

## 5. Conclusions

This meta-analysis integrates existing studies to compare the effectiveness of CWI and CWI + Other interventions in post-exercise fatigue recovery. The results indicate that both interventions are effective in reducing DOMS. While CWI + Other showed a trend toward a larger effect, particularly in athletes, where the relief was more pronounced, no statistically significant difference was found between the two approaches overall. Additionally, CWI + Other significantly reduced CRP levels, whereas CWI only demonstrated a reduction in CRP when the water temperature exceeded 10 °C. Future studies should focus on increasing the sample sizes, optimizing research designs to reduce bias and exploring the interactions between recovery outcomes and factors such as exercise type, age and gender. Furthermore, more research is needed to identify the optimal combinations of interventions within the CWI + Other approach to achieve precise and personalized post-exercise fatigue recovery strategies.

## Figures and Tables

**Table 1:** Characteristics of the included studies.

								Intervention		Mean Age, Years	
Study, Year	Study Type	Number of Cases (E/C)	Sex (M/F)	Profession	Exercise Protocol	Outcome Measures	Time of Measure	E	C	E	C
Bartley, 2021 [22]	RCT	15/14	22/7	Athletes	Ironman game	CK DOMS	Baseline 24 h 48 h	CWI 10 °C 12 min	PAS	42.5 ± 2.3	46.0 ± 3.2
Sánchez-Ureña, 2017 [20]	Randomized, crossover	10/10	10/0	Athletes	Basketball technical and tactical skills training	CMJ DOMS	Baseline 24 h 48 h	CWI 12 °C 12 min	PAS	14 ± 0.4	14 ± 0.4
Chow, 2018 [23]	RCT	13/22	13/22	Non-athletes	HIIT	CMJ	PRE POST	IWI 5 °C 1 min	PAS	22.9 ± 2.4	20.7 ± 2.4
Duffield, 2014 [24]	Randomized, crossover	8/8	8/0	Athletes	Tennis training	CMJ DOMS	PRE POST	CWI 11 °C 15 min + compression garments	PAS	20.9 ± 3.6	20.9 ± 3.6
De Brito, 2022 [25]	Randomized, crossover	14/14	/	Non-athletes	10 RM	CK CRP	Baseline 2 h	CWI 15 °C 10 min + vitamin C/E	PAS	26.2 ± 5	26.2 ± 5
Pesenti-Tofalini, 2020 [26]	RCT	5/5	10/0	Athletes	Soccer match	CK	Baseline POST 24 h 48 h	CWI 10 °C 10 min	PAS	17.6 ± 0.54	16.8 ± 0.83
Crowther, 2017 [27]	RCT	34/34	34/0	Non-athletes	Simulated team game	CMJ DOMS	Baseline POST 24 h 48 h	CWI 15 °C 14 min + low-intensity cyclic leg movement	PAS	27 ± 6	27 ± 6
Tavares, 2020 [28]	RCT	7/6	/	Athletes	Regular volleyball training and resistance training	CMJ	Baseline POST training camp	CWI	PAS	19.2 ± 0.8	19.0 ± 1.3
Glasgow, 2014 [29]	RCT	10/10	/	Non-athletes	Three sets of eccentric hamstring contractions to fatigue	CK DOMS	Baseline 24 h 48 h	CWI 10 °C 10 min	PAS	/	/
Difranco, 2022 [30]	RCT	8/10	18/0	Athletes	A competitive trail marathon run	CK CRP	Baseline 24 h 48 h	CWI 8 °C 10 min + cherry juice	Placebo + PAS	42.7 ± 4.7	40.6 ± 7.2
Rupp, 2012 [31]	RCT	12/10	13/9	Athletes	YO-YO test	CMJ DOMS	Baseline 24 h 48 h	CWI 12 °C 15 min	PAS	19.8 ± 1.1	19.8 ± 1.1
Kositsky, 2020 [32]	RCT	5/5	10/0	Athletes	Drop jumps	CK DOMS	Baseline 24 h 48 h	CWI 10 °C 20 min	PAS	19.4 ± 0.9	18.4 ± 0.5
Krueger, 2020 [33]	RCT	9/9	18/0	Athletes	Hockey game	CK DOMS	Baseline 24 h 48 h	CWI 6.4 ± 0.8 °C 5 min	PAS	16.6 ± 0.6	16.6 ± 0.6
Leeder, 2019 [34]	RCT	11/10	21/0	Athletes	Loughborough Intermittent Shuttle Test	CK CRP	PRE POST	CWI 14 °C 14 min	PAS	20 ± 2	19 ± 1
Machado, 2017 [35]	RCT	20/20	40/0	Non-athletes	Eccentric exercise	CK DOMS	Baseline 24 h 48 h	CWI 9 °C 15 min	PAS	21.2 ± 2.0	20.4 ± 1.8
Takeda, 2014 [36]	Randomized, crossover	10/10	20/0	Athletes	Rugby simulation training	CK CMJ	Baseline 24 h	CWI 15 °C 10 min	PAS	/	/
Nasser, 2023 [37]	Randomized, crossover	12/12	24/0	Athletes	Loughborough Intermittent Shuttle Test	CMJ DOMS	Baseline 24 h 48 h	CWI 11 °C 15 min	PAS	/	/
Angelopoulos, 2022 [38]	RCT	15/15	30/0	Athletes	Plyometric fatigue protocol	CK DOMS	Baseline POST 24 h 48 h	20 min massage + CWI 10 °C 10 min	PAS	/	/
Pointon, 2012a [39]	RCT	10/10	20/0	Athletes	Loughborough Test (HIIT)	CK CRP	Baseline POST 2 h 24 h	CWI 9 °C 9 min + 1 min rest × 2	PAS	/	/
Pointon, 2012b [40]	Randomized, crossover	10/10	10/0	Athletes	Intermittent-sprint exercise	CK CPR	Baseline POST 2 h 24 h	CWI 9 °C 9 min + 1 min rest × 2	PAS	/	/
Schimpchen, 2017 [41]	Randomized, crossover	7/7	7/0	Athletes	Weightlifting training	CK	Baseline 24 h	CWI 12–15 °C 10 min	PAS	/	/
Siqueira, 2018 [42]	RCT	14/15	29/0	Non-athletes	Drop jumps	CK DOMS CRP	Baseline 24 h 48 h	CWI 10 °C 20 min	PAS	20.5 ± 1.4	19.9 ± 1.4
Maruyama, 2019 [43]	Randomized, crossover	10/10	10/0	Non-athletes	Eccentric exercise	CK DOMS CRP	Baseline 3 h 24 h	CWI 15 °C 15 min + lower-body compression garment	PAS	/	/
Wiewelhove, 2018 [44]	RCT	11/12	23/0	Non-athletes	Half-marathon race	CK CMJ DOMS CRP	Baseline POST 24 h	CWI 15 °C 15 min	PAS	31.5 ± 10.2	31.3 ± 12.3

E, experimental group; C, control group; M, male; F, female; RCT, randomized controlled trial; HIIT, high-intensity interval training; CK, creatine kinase; CMJ, countermovement jump; DOMS, delayed-onset muscle soreness; CRP, C-reactive protein; CWI, cold-water immersion; PAS, passive recovery. The “a” and “b” in Pointon 2012 are used to distinguish between two different articles published by the same author in the same year.

## Data Availability

The dataset is available upon request from the authors.

## Supplementary Materials

The following supporting information can be downloaded at: https://www.mdpi.com/article/10.3390/life15081205/s1, File S1: Summary of Database Search Terms; File S2: Figures S1–S3.
