# Supplementary material for: Comparison of the Effects of Cold-Water Immersion Applied Alone and Combined Therapy on the Recovery of Muscle Fatigue After Exercise: A Systematic Review and Meta-Analysis
Source: Life (Basel). 2025 Jul 28;15(8):1205. doi: 10.3390/life15081205 (PMC12387994; doi:10.3390/life15081205)
Supplement: Supplementary file 1 [file life-15-01205-s001.zip › Supplemental File S1.pdf]

Summary of Database Search Terms

1.PubMed

#1 "cold water immersion"[Title/Abstract] OR "CWI"[Title/Abstract] OR "cold bath"[Title/Abstract] OR "hydrotherapy"[Title/Abstract] OR "combined therapy"[Title/Abstract] OR "adjunct therapy"[Title/Abstract] OR "multimodal recovery"[Title/Abstract] OR "mixed method recovery intervention"[Title/Abstract]

Results: 22,950

#2 "creatine kinase"[Title/Abstract] OR "ck"[Title/Abstract] OR "rating of perceived exertion"[Title/Abstract] OR "RPE"[Title/Abstract] OR "blood lactate"[Title/Abstract] OR "lactate"[Title/Abstract] OR "delayed onset muscle soreness"[Title/Abstract] OR "DOMS"[Title/Abstract] OR "maximal voluntary contraction"[Title/Abstract] OR "MVC"[Title/Abstract] OR "countermovement jump"[Title/Abstract] OR "CMJ"[Title/Abstract]

Results: 211,590

#3 "randomized controlled trial"[Title/Abstract] OR "randomized"[Title/Abstract] OR "RCT"[Title/Abstract]

Results: 763,266

#4 #1 AND #2 AND #3

Results: 79

2.Embase

#1 'cold water immersion':ab,ti OR 'CWI':ab,ti OR 'cold bath':ab,ti OR 'hydrotherapy':ab,ti OR 'combined therapy':ab,ti OR 'adjunct therapy':ab,ti OR 'multimodal recovery':ab,ti OR 'mixed-method recovery intervention':ab,ti

Results: 31,070

#2 'ck':ab,ti OR 'RPE':ab,ti OR 'rating of perceived exertion':ab,ti OR 'blood lactate':ab,ti OR 'lactate':ab,ti OR 'delayed onset muscle soreness':ab,ti OR 'DOMS':ab,ti OR 'maximal voluntary contraction':ab,ti OR 'MVC':ab,ti OR 'countermovement jump':ab,ti OR 'CMJ':ab,ti

Results: 259,676

#3 'randomized controlled trial':ab,ti OR 'randomized':ab,ti OR 'RCT':ab,ti

Results: 1,089,959

#4 #1 AND #2 AND #3

Results: 72

3.Web of Science
#1 TS=(cold water immersion) OR TS=(CWI) OR TS=(cold bath) OR TS=(hydrotherapy) OR TS=(combined therapy) OR TS=(adjunct therapy) OR TS=(multimodal recovery) OR TS=(mixed-method recovery intervention)

Results: 159,476

#2 TS=(creatine kinase) OR TS=(ck) OR TS=(rating of perceived exertion) OR TS=(RPE) OR TS=(blood lactate) OR TS=(lactate) OR TS=(delayed onset muscle soreness) OR TS=(DOMS) OR TS=(maximal voluntary contraction) OR TS=(MVC) OR TS=(countermovement jump) OR TS=(CMJ)

Results: 138,666

#3 TS=(randomized controlled trial) OR TS=(randomized) OR TS=(RCT)

Results: 676,486

#4 #1 AND #2 AND #3

Results: 265

4. Cochrane
#1 (cold water immersion):ti,ab,kw OR (CWI):ti,ab,kw OR (cold bath):ti,ab,kw OR (hydrotherapy):ti,ab,kw OR (combined therapy):ti,ab,kw OR (adjunct therapy):ti,ab,kw OR (multimodal recovery):ti,ab,kw OR (mixed-method recovery intervention):ti,ab,kw

Results: 115,967

#2 (creatine kinase):ti,ab,kw OR (ck):ti,ab,kw OR (RPE):ti,ab,kw OR (rating of perceived exertion):ti,ab,kw OR (blood lactate):ti,ab,kw OR (lactate):ti,ab,kw OR (delayed onset muscle soreness):ti,ab,kw OR (DOMS):ti,ab,kw OR (maximal voluntary contraction):ti,ab,kw OR (MVC):ti,ab,kw OR (countermovement jump):ti,ab,kw OR (CMJ):ti,ab,kw

Results: 25,736

#3 (randomized controlled trial):ti,ab,kw OR (randomized):ti,ab,kw OR (RCT):ti,ab,kw

Results: 1,207,626
#4 #1 AND #2 AND #3

Results: 965

5. EBSCO
#1 AB (cold water immersion) OR AB (CWI) OR AB (cold bath) OR AB (hydrotherapy) OR AB (combined therapy) OR AB (adjunct therapy) OR AB (multimodal recovery) OR AB (mixed-method recovery intervention)

Results: 14,500

#2 AB (creatine kinase) OR AB (ck) OR AB (RPE) OR AB (rating of perceived exertion) OR AB (blood lactate) OR AB (lactate) OR AB (delayed onset muscle soreness) OR AB (DOMS) OR AB (maximal voluntary contraction) OR AB (MVC) OR AB (countermovement jump) OR AB (CMJ)

Results:3221

#3 AB (randomized controlled trial) OR AB (randomized) OR AB (RCT)

Results:151036

#4 #1 AND #2 AND #3
Results: 121
