# Supplementary figures and images for: Comparison of the Effects of Cold-Water Immersion Applied Alone and Combined Therapy on the Recovery of Muscle Fatigue After Exercise: A Systematic Review and Meta-Analysis
Source: Life (Basel). 2025 Jul 28;15(8):1205. doi: 10.3390/life15081205 (PMC12387994; doi:10.3390/life15081205)

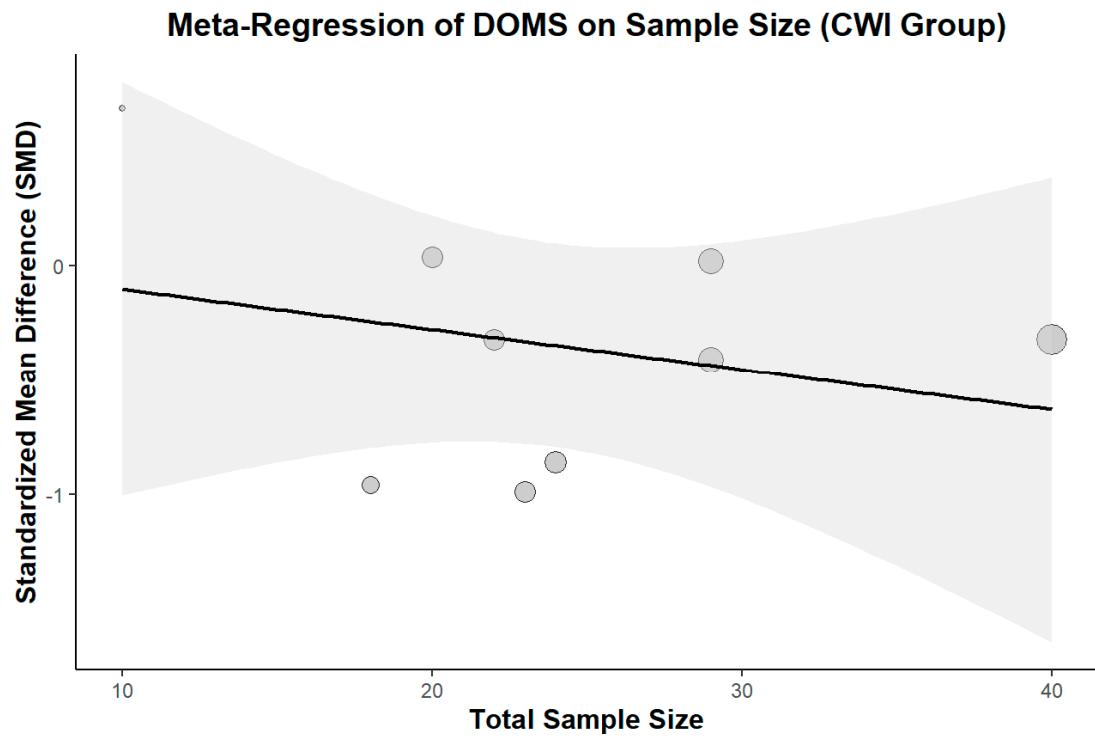

Supplemental Figure S 1.

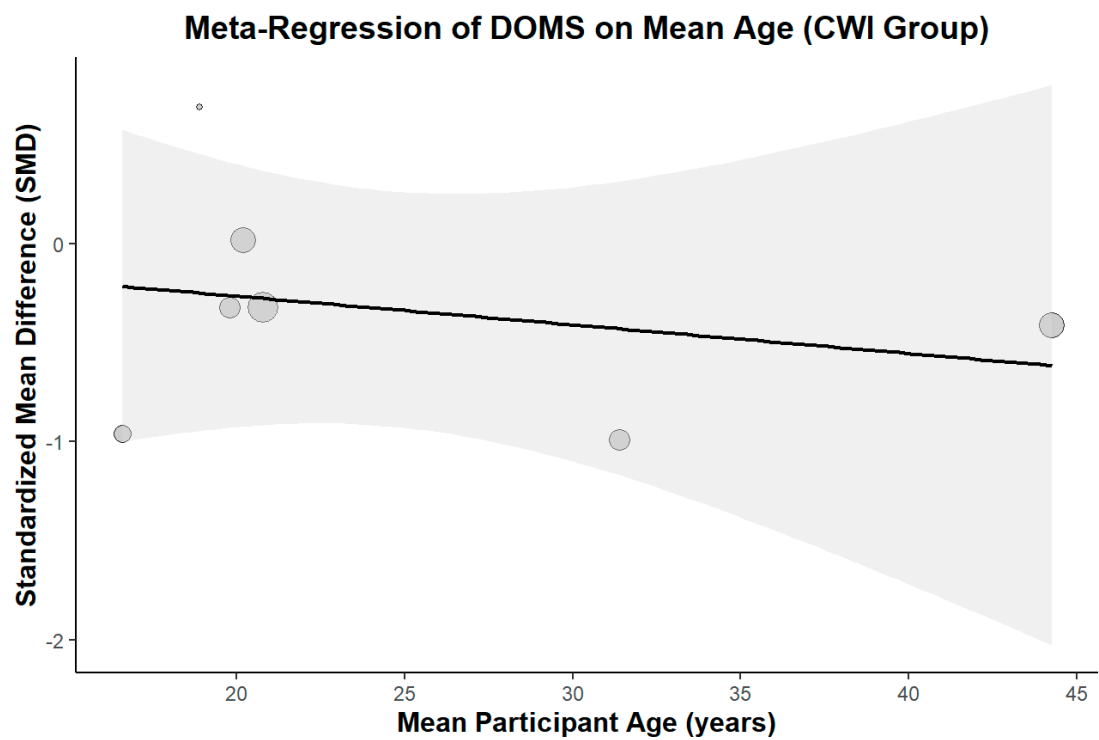

Supplemental Figure S 2.

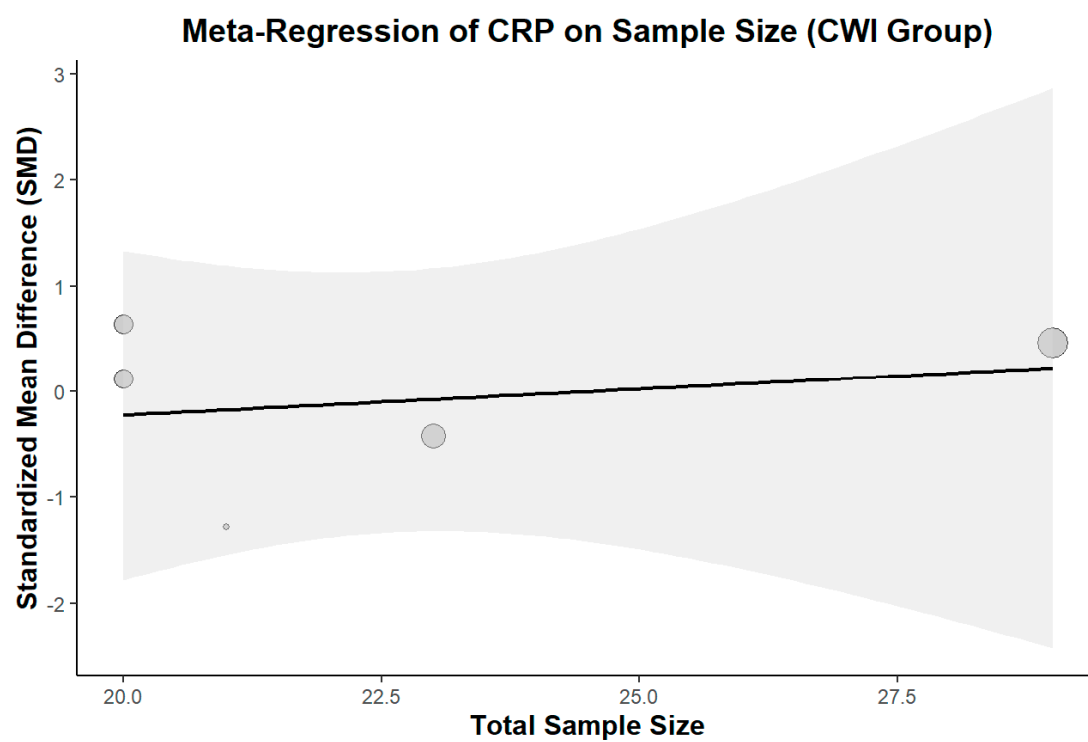

Supplemental Figure S 3.

Supplement: Supplementary file 1 [file life-15-01205-s001.zip › Supplemental File S2.pdf]
